# *Challenging the status quo:* results of an acceptability and feasibility study of hypertensive disorders of pregnancy (HDP) management pathways in Indonesian primary care

**DOI:** 10.1186/s12884-021-03970-8

**Published:** 2021-07-14

**Authors:** Fitriana Murriya Ekawati, Ova Emilia, Jane Gunn, Sharon Licqurish, Phyllis Lau

**Affiliations:** 1 Department of Family and Community Medicine, Universitas Gadjah Mada, Yogyakarta, Indonesia; 2 Department of General Practice, University of Melbourne, Parkville, Victoria Australia; 3 Department of Obstetrics and Gynaecology, Universitas Gadjah Mada/Sardjito Hospital, Yogyakarta, Indonesia; 4 School of Nursing and Midwifery, Monash University, Clayton, Victoria Australia; 5 Department of General Practice, Western Sydney University, New South Wales, Australia

**Keywords:** Hypertensive disorders of pregnancy, Preeclampsia, Indonesia, Management, Implementation, Barriers, Acceptability, Feasibility, LMICs, Primary care

## Abstract

**Background:**

Hypertensive disorders of pregnancy (HDP) are the leading cause of maternal mortality in Indonesia. Focused HDP management pathways for Indonesian primary care practice have been developed from a consensus development process. However, the acceptability and feasibility of the pathways in practice have not been explored. This study reports on the implementation process of the pathways to determine their acceptability and feasibility in Indonesian practice.

**Methods:**

The pathways were implemented in three public primary care clinics (Puskesmas) in Yogyakarta province for a month, guided by implementation science frameworks of Medical Research Council (MRC) and the Practical Robust Implementation and Sustainability Model (PRISM). The participating providers (general practitioners (GPs), midwives, and nurses) were asked to use recommendations in the pathways for a month. The pathway implementation evaluations were then conducted using clinical audits and a triangulation of observations, focus groups (FGs), and interviews with all of the participants. Clinical audit data were analysed descriptively, and qualitative data were analysed using a mix of the inductive-deductive approach of thematic analysis.

**Results:**

A total of 50 primary care providers, four obstetricians, a maternal division officer in the local health office and 61 patients agreed to participate, and 48 of the recruited participants participated in evaluation FGs or interviews. All of the providers in the Puskesmas attempted to apply recommendations from the pathways to various degrees, mainly adopting preeclampsia risk factor screenings and HDP monitoring. The participants expressed that the recommendations empowered their practice when it came to HDP management. However, their practices were challenged by professional boundaries and hierarchical barriers among health care professionals, limited clinical resources, and regulations from the local health office. Suggestions for future scale-up studies were also mentioned, such as involving champion obstetricians and providing more patient education toolkits.

**Conclusion:**

The HDP management pathways are acceptable and feasible in Indonesian primary care. A further scale-up study is desired and can be initiated with investigations to minimise the implementation challenges and enhance the pathways’ value in primary care practice.

**Supplementary Information:**

The online version contains supplementary material available at 10.1186/s12884-021-03970-8.

## Background

Hypertensive disorders of pregnancy (HDP) are the second-highest cause of global maternal mortality [1]. Diagnosis criteria for HDP include ranges of hypertensive conditions in pregnancy, such as chronic hypertension, gestational hypertension, and preeclampsia. The disorders are experienced by up to 10% of pregnant women worldwide [1, 2], and related complications can lead to maternal deaths, which mostly occur in low- and middle-income countries (LMICs) [1, 3]. In Indonesia, HDP causes about a thousand maternal deaths each year and a high number of infant morbidity, as many women with HDP have to deliver their babies prematurely [4, 5].

Despite the significant impact on maternal mortality caused by HDP, unfortunately there are limited guidelines on HDP management available in Indonesian primary care [6]. The existing HDP guidelines in the setting only recommend general practitioners (GPs) to refer pregnant women to hospitals. However, more detailed on the screening, diagnostics, and HDP procedures before referrals are hardly mentioned [7, 8]. Therefore, it is not surprising that many women already present with severe HDP conditions at the hospitals, as many do not receive appropriate management in primary care, such as antihypertensive medications and the maintenance of adequate maternal and foetal wellbeing [9, 10].

This paper reports the final phase of a larger research project which aimed to develop HDP management pathways for Indonesian primary care [6]. Recommendations included in the HDP pathways were informed by results of a review of international HDP guidelines and preliminary interviews with Indonesian key stakeholders [11, 12]. The recommendations then went through a consensus development process using Delphi technique to explore the experts' agreement for the readiness of the recommendations to be used in Indonesian primary care settings [6, 13, 14]. The aim of this final phase was to determine the acceptability and feasibility of the pathways in Indonesian primary care practice. Specific objectives covered in this phase were to explore Indonesian primary care providers’ experience of using the pathways, including implementation barriers and facilitators for the pathways in practice settings.

## Methods

### Theoretical Frameworks

Two implementation science frameworks were used to guide the study. The first framework, the Medical Research Council (MRC) [15], guided the study stages to determine acceptability and feasibility of the pathways before their implementation in more extensive primary care settings [6]. The second framework, the Practical Robust Implementation and Sustainability Model (PRISM) [16], was used to guide the development of guiding questions in the implementation evaluation, as well as to guide selections of recipients of the pathways/participants in the study. According to PRISM, they were those who used or were impacted by the pathways, such as GPs, nurses, midwives, patients, obstetricians, and local health officers [6, 16].

### Design

This study applied a mixed-methods design informed by MRC [15] and PRISM [16]. The pathway implementation was conducted from July–November 2019 and consisted of three implementation stages:

- Stage 1 – Capacity building. Pre-implementation seminars were held at each Puskesmas to provide participants with information regarding the HDP pathways.
- Stage 2 – Implementation. The developed HDP pathways were implemented in three Puskesmas for a month. The Puskesmas providers were asked to use and apply recommendations in the pathways in their routine antenatal care (ANC).
- Stage 3 – Implementation evaluation. The evaluation was conducted following a one-month implementation using a triangulation approach of clinical audits, observations, focus groups (FGs), and interviews with primary care providers, as well as interviews with patients, obstetricians, and local health officers.

### Study setting

This study took place in three Puskesmas in Bantul District, Yogyakarta Province, Indonesia. Puskesmas are government-mandated community primary health care clinics located across Indonesia that provide individual health consultations, surveillance and public health programs. They are the backbone of the Indonesian primary care service, ensuring the availability of the service across Indonesian provinces [17]. Yogyakarta itself has 121 Puskesmas and 27 of them are located in Bantul district [18]. Bantul was selected as the setting of the study for its high number of maternal mortality cases in 2019. From 36 maternal deaths in Yogyakarta, third of the cases were from Bantul and around a third of the total cases in Yogyakarta were caused by preeclampsia [18, 19].

Regarding the three Puskesmas: Puskesmas 1 is a satellite clinic located in a rural area in the district. Patients accessing Puskemas 1 tend to be of low socioeconomic status, and include workers such as farmers and labourers. Puskesmas 2 is located in a more metropolitan area, close to the provincial capital, and provides care to wealthier populations compared to Puskesmas 1. Puskesmas 3 is located in a rural coastal area and most of its patients are farmers or fishermen. Most antenatal care (ANC) procedures in the three Puskesmas are covered by *Jaminan Kesehatan Nasional* (JKN) as the Indonesian public insurance [20] or *Jaminan Kesehatan Daerah (Jamkesda) as* the local district-level insurance [21].

### Interventions

**Fig. 1:**
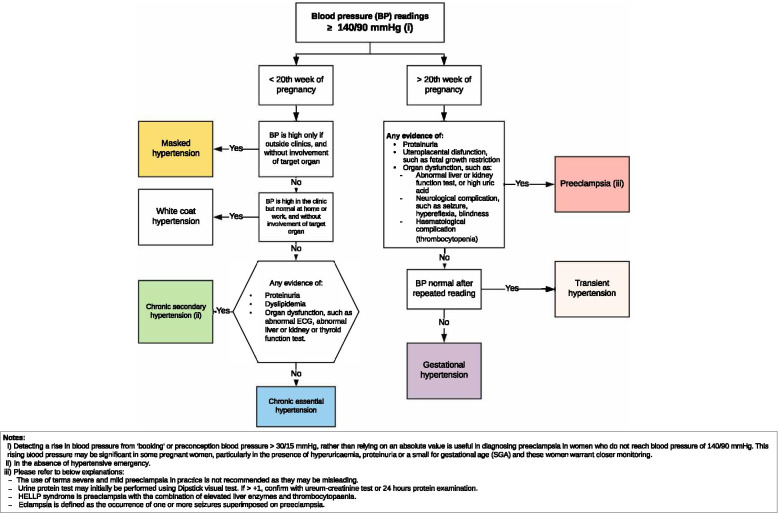
HDP diagnosis flowchart [22]

**Fig. 2:**
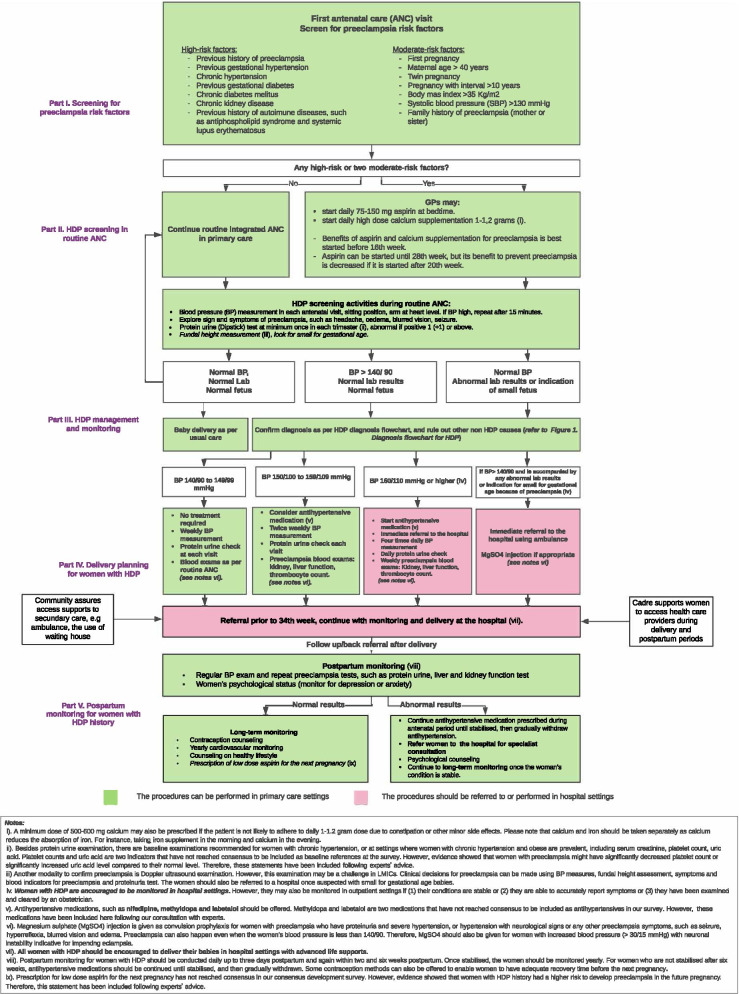
Hypertensive disorders of pregnancy (HDP) management pathway used in the study, developed from the consensus development process [22]

**Fig. 3:**
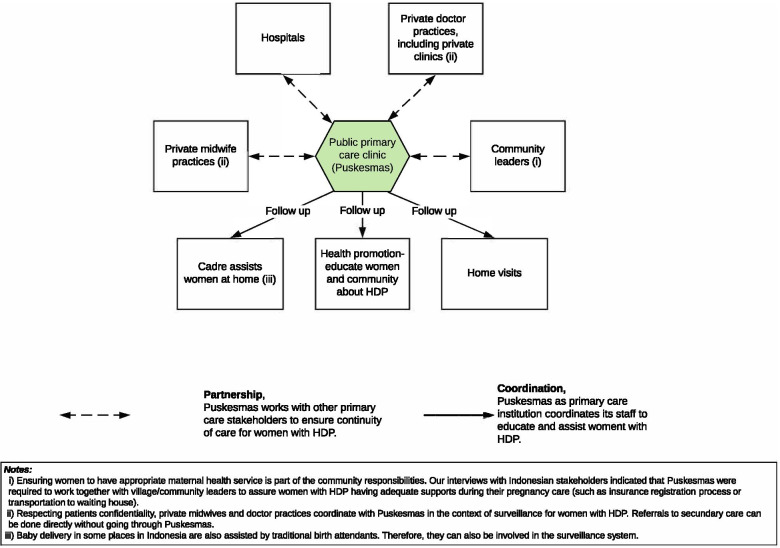
Surveillance pathway for women with HDP in Indonesian primary care [22]

The primary interventions implemented in the study were the developed HDP management pathways for Indonesian primary care [22]. They consisted of: (i) an HDP diagnosis flowchart (Fig. 1); (ii) an HDP management pathway (Fig. 2); and (iii) an HDP surveillance pathway (Fig. 3) [22]. The participants were then also provided with multifaceted intervention toolkits, such as:

- Educational tools. Each Puskesmas received up to 20 training modules containing a detailed explanation of the HDP pathways and two HDP management pathways posters (see Fig. 2).
- Reminder tools. Primary care provider participants received stickers, pens, andlanyards, and patient participants received mugs, notebooks, and stickers explaining essential information on HDP management.
- A patient examination tool. Primary care provider participants received a checklist of high and moderate clinical preeclampsia risk-factors to enable a more practical patient screening in Puskesmas.

### Recruitment

#### Puskesmas

The three participating Puskesmas were identified from the first author’s networks and were approached following an initial consultation with the maternal health division in the local health office. Formal invitations, including the plain language statement (PLS) and consent form, were also sent to head of each Puskesmas for their participation approval.

#### Clinicians and the health officer

Recruitment of the primary care providers, such as GPs, nurses, and midwives, was conducted face-to-face at each Puskesmas after obtaining research approval from the head of each Puskesmas. Obstetricians working within the Puskesmas’ networks and local health officers were invited to participate via phone, short message service (SMS) [23], and WhatsApp messenger [24]. All participants were provided with PLS and consent form of the study, and they had opportunities to ask questions before consenting.

#### Patients

Patient recruitment was conducted in waiting rooms in each Puskesmas. The women were asked politely whether they visited Puskesmas for their ANC and whether they were interested to participate in the study. They were provided with the study PLS and consent form, and were able to ask any questions before consenting. They were also informed that they might receive different treatments, medications, or tests compared to their usual care, and that their ANC consultations may be observed and recorded. At the end of the implementation period, women who attended a minimum of two ANC visits in the Puskesmas were invited to participate in the evaluation interviews.

### Data collection

The data collection for the pathway implementation evaluation consisted of clinical audits, observations of GP/midwife-patient consultations, interviews, and focus groups (FGs). The clinical audits included the number of women attending ANC visits in the Puskesmas; the number of women who received preeclampsia screenings using the checklist of clinical risk factors; the number of dipstick tests and amounts of antihypertensive medication prescribed; and the number of HDP women being referred to hospitals in a month. These data were extracted from the Puskesmas electronic information system at the end of the implementation period.

During the observation, the first author (observer) used the ‘observer as participants’ approach [25]. It is an approach that provides flexible opportunities for the observer to be involved in the participants' activities and have some interactions with them [25]. The observations were made after midwives and GPs in the Puskesmas notified the first author when they welcomed HDP patients or those with preeclampsia risk factors who consented to participate in the study. The participants were aware of the observation, and the observation video recordings were conducted with their permission. The observations were conducted twice, for an hour each, with 30 min of video recording (a total of 60 min recording) in each Puskesmas. Any significant but unrecorded observations were also noted in the first author’s fieldwork notes.

At the evaluation interviews/FGs, primary care provider participants were asked about their experience using the pathways, including implementation barriers and facilitators of the pathways in primary care. Obstetricians and local health officer participants were asked about their experience of receiving referrals for patients with HDP cases, including their views and suggestions for the future scale-up study. All FGs and interviews were conducted in Bahasa Indonesia by the first author to aid natural discussions with the participants and were conducted at the participating Puskesmas or by telephone, based on the participants’ preference. The core interview/FG questions used in this evaluation have previously been published in a study protocol paper [6] and the complete interview/FG questions in English have also been attached in Supplementary File 1.

### Data analysis

Clinical audit data obtained in the study were analysed descriptively using Microsoft Excel software [26]. The numbers of women who visited for ANC in each Puskesmas, and who received any HDP screening, laboratory examinations and/or medications, were recorded and counted for their mean.

Qualitative data obtained from the observations, FGs, and interviews were analysed thematically. The thematic analysis process was performed as follows: (i) the FGs and interviews were transcribed and translated into English. (ii) Observation videos were described in English, viewed, and noted for any significant scenes; field notes taken during observations and the implementation were also translated into English. (iii) All transcripts, video descriptions and field notes were imported into Nvivo software [27] and were repeatedly read for data familiarisation. (iv) The data were then coded for any notable quotes, scenes, or notes and the codes were then grouped into overarching themes and subthemes according to PRISM domains [16] (deductive approach) as well as to elicit new themes (inductive approach) [15, 16]. The co-authors also validated the coding process and the results were discussed until coding consensus was agreed. Reporting of this study follows the Standards for Quality Improvement Reporting Excellence (SQUIRE) checklist [28] in Supplementary File 2.

### Language validation

All PLSs, consent forms, implementation toolkits, and FG/interview questions were initially created in English, translated into Bahasa Indonesia, and then back-translated into English by the first author. An Indonesian native speaker then validated the translation of PLSs and consent forms to ensure language validation. All FG/interview transcripts in Bahasa Indonesia were also translated into English by the first author to aid discussion among the project investigators. A quarter of the transcripts were back-translated into Bahasa Indonesia by another native Indonesian speaker to ensure translation validation [29].

## Results

### Participants

**Fig. 4:**
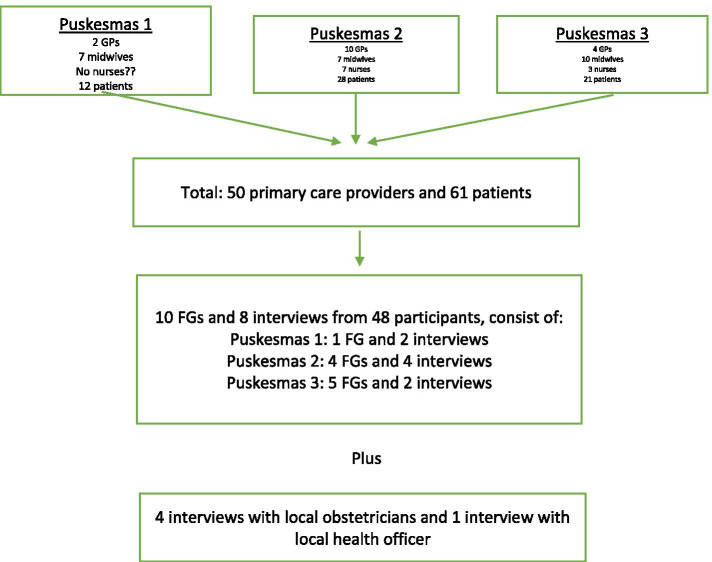
Flowchart of participants recruitment to focus groups (FGs) and interviews

Fifty primary care providers (16 GPs, 24 midwives, and ten nurses) from three participating Puskesmas, four obstetricians, one local health officer (a maternal health division officer), and 61 patients agreed to participate in this study (see Fig. 4). The majority of the clinicians were female (n = 46) and aged 20–40 years (n = 32), while the majority of patients were aged 20–30 years (n = 35), were housewives (n = 41), and had been registered in the Puskesmas for less than five years (n = 38). However, only 23 out of 61 patients provided their phone numbers for follow-up interview/FG arrangements. The participants’ recruitment flowchart and characteristics are presented in Fig. 4 and Table 1 and 2.

**Table 1:** Participants characteristic (health care providers)

Details	Number (in total)
Occupation	
GPs	16
Midwives	24
Nurses	10
Obstetricians	4
Health officer	1
Gender	
Male	9
Female	46
Age range	
20–30	13
30–40	19
40–50	16
> 50 years old	7
Practice experience	
< 5 years	14
6–10 years	8
11–15 years	12
16–20 years	8
> 20 years	13

**Table 2:** Participants characteristic (patients)

Details	Number (total)
Had HDP history	5
Age ranges (years)	
20–30	35
30–40	26
Been a patient at the clinic (years)	
0–5	38
6–10	13
11–15	1
16–20	2
> 20 years	7
Residence	
Rural	22
Urban	39
Occupation	
Housewife	41
Casual worker	17
Civil servant	1
Lecturer	1
School teacher	1
Highest education level achieved	
Primary school	2
High school	44
University	15
Family income level per month	
< IDR^a^ 2.5 million	39
IDR 2.5–5 million	20
> IDR 5 million	2
Provide details for interviews	
Yes	23
No	38

^a^*IDR* Indonesia Rupiah

### Results of the pathway implementation evaluation

#### Clinical audits

During the one-month intervention period, GPs, midwives, and nurses in three Puskesmas attempted to apply recommendations in the HDP pathways in various degrees (see Table 3). The most commonly applied recommendations were the preeclampsia risk factors screening (n = 141) and dipstick tests examination (n = 78). Some procedures of HDP management, such as the administration of aspirin (n = 24) and nifedipine prescriptions (n = 8), were also conducted by the Puskesmas. Puskesmas 1 had the highest percentage of preeclampsia risk factor screenings (82.14%), but Puskesmas 2 had the highest number of ANC visits and of low-dose aspirin prescriptions for preeclampsia prevention. Puskesmas 1 and 2 did not perform liver and kidney function tests due to limited clinical resources available in the clinics. However, all Puskesmas referred similar numbers of HDP patients to the hospital.

**Table 3:** Clinical audit data presenting number of women receive any examination or procedures according to the pathways

Items (in a month pilot implementation)	Number of pregnant women (n)				
	Puskesmas 1	Puskesmas 2	Puskesmas 3	Total	Mean
Visiting women	112	267	197	576	192
Women screened with preeclampsia risk factors	92	15	34	141	47
Women with one high-risk or at least two moderate risk-factors for preeclampsia	5	20	10	35	11.7
Women tested with dipstick protein urine	13	51	14	78	26
Women went for liver and kidney function tests	0	0	2	2	0.7
Women received aspirin as preeclampsia prophylaxis	2	17	5	24	8
Women received antihypertensive medication	5	0	3	8	2.7
Number of women referred to the hospitals from HDP	2	3	2	7	2.3

#### Observations

A total of six observations were conducted, and each Puskesmas had an hour of video-recording. Some notable observations were noted and are described below. It was challenging at the beginning of the implementation to engage Puskesmas staff because they had many patients and were busy with community outreach programs or *Pos Pelayanan Terpadu* (*Posyandu*) in the afternoon [30]. For this reason, a GP in Puskesmas 3 requested a risk-factor checklist to enable a more practical method of preeclampsia risk-factor screening in their patient encounters. Besides that, Puskesmas 2 already prescribed low-dose aspirin for women with a history of pregnancy hypertension, albeit before the study, it was prescribed only for a few days’ duration, and the patients were referred to the obstetricians. Meanwhile, the other two Puskesmas had only just initiated aspirin prophylaxis at the time of this study. Puskesmas 2 also had a visiting obstetrician who promoted aspirin prescription. All Puskesmas also provided home visits for pregnant women with HDP or preeclampsia risk factors, and these visits were embedded in their Posyandu schedules.

#### Focus groups (FGs) and interviews

A total of 43 clinicians and five patients participated in ten FGs and 13 interviews at the end of the implementation period. One FG and two interviews were conducted with primary care clinicians at Puskesmas 1; four FGs and one interview were conducted with clinicians at Puskesmas 2; and five FGs with clinicians at Puskesmas 3. Other interviews conducted consisted of four with obstetricians, one with the local health officer, and five with patients. All FGs consisted of two to six participants, and all participants only participated in one FG or interview each. The length of the FGs/interviews ranged from 15 to 60 min. All FGs/interviews were recorded and transcribed except for four interviews (three with obstetricians and one with a patient) due to the participants’ refusal for recording. Significant quotes during these four interviews were noted and presented afterward to the participants for content validation.

#### Themes arising from the analysis of observations, focus groups, and interview transcripts

Four themes were elicited from video-observation descriptions, and from FG and interview transcripts. The themes reflect successful impacts of the pathways, as well as notable barriers during the implementation of the pathways in primary care practice. Quotes supporting the themes are presented in Table 4.

**Table 4:** Quotes for each theme

Theme	Subthemes	Quotes
Empowerment	Well-designed intervention	“*The pathways are easy, not that difficult and they focus on preeclampsia” (GP 1, Interview1)* *“Your toolkits are helpful, the checklist I think is the most important toolkit, the module is good to improve our knowledge” (Midwife 2, FG 8)* *“The toolkits are really good. The information listed in the mugs and stickers is easy to understand and I used those for my Whatsapp status” (Patient 5, Interview 13)* *“The pathways are very clear and succinct compared to the routine ANC form, that is more detailed. The ANC form has many coloumns to fill, and we got confused at the end as the conclusion is meaningless, only whether the patient has an infection or not, and no further follow up” (Interview 1, GP)*
	Empowerment	“*We have been warned for referring many women and we have massive ‘red lines’ from the JKN system, but I am thankful that we now have this guidance for aspirin prescription and to monitor their conditions. We feel that we are helped” (Midwife 5, FG 4)*
Hierarchy	Between nurses, midwives and GPs	*“We could not prescribe medicines because it is not our responsibility. If there are any pregnancy complications, it should be the midwives who can follow up and do the standard operating procedures (SOP)” (Nurse 1, FG 10)*
	Between GPs and patients	*“I think all patients are so obedient to our advice. I found that the patients are often defying doctors like Gods. So the doctors are everything and they are so obedient”* *“I agree, that when we give the prescription, they also agree to take it. No further questions” (GP 2 and 4, FG 2)* (notes: However, these quotes contradict with results of our observations that some patients seem afraid with their doctors)
	Between GPs and specialists	*“I prefer to consult (the obstetrician) first. I am afraid if I will get audited for making a mistake. Then I would be asked about this and that. But often, the hospital advice is also very little. A specialist stopped our aspirin prescription last week. He only prescribed a few tablets and provided no further advice for our patients (and the GPs could not discuss the patient management)” (GP 1, FG 3)* *Aspirin should be given under 16 weeks to prevent preeclampsia and indeed, it is preventive management that I think should be conducted more aggressively because of our high maternal mortality rate. Overdiagnosis is also good. For example, we recommend that women who have doubtful (dipstick) proteinuria to be seen as a positive result. (Therefore,) Primary care should also upgrade their knowledge regarding the patients’ condition and (including preeclampsia prevention) in the community. For example, how is the community consumption level for calcium and iron” (Interview 5, Obstetrician 2)* *“The risk factors screening is good, GPs can perform screening and refer high-risk patients to us, then us (obstetricians) can do further tests and start Aspirin if necessary” (Interview 3, Obstetrician)*
	Between primary care providers and local health office	*“Currently, we are under the maternal emergency policy and we are closely monitored for pregnancy complication management. This policy somehow makes us scared and paranoid if we are get audited if the patients do not see the specialist in the first place. Therefore, we liked to refer women with complications to the hospital” (GP 1, FG 3)*
Clinical resource		*“We now still have the reagents so that I am able just to check SGOT SGPT and it is free for the patients, but later we might have limited reagents and we should consider that too” (GP 1, FG 9)* “*if we have to provide aspirin for nine months of the pregnancy, then we have to also order that from the local health office” (GP1, FG 7)*
Direction	Uniformity	*“I think further research to scale-up the implementation of the pathways will be good. First, it (the pathway) is beneficial in this Puskesmas. if that is also applied in other Puskesmas, that will be better so that we are in one rhyme with them*” (Midwife 2, FG 8)
	Conformity with current medical record system	*“We don’t really use the diagnosis of transient or masked hypertension, I know there are some conditions like that, but if we enter them in the medical record system, they don’t appear as a clinical diagnosis” (Interview 5, Obstetrician)*
	Supporting policy	*“We do not have a legal umbrella (policy from the local health officer) yet. Even though we have seen patients with preeclampsia also patients with moderate and high-risk factors and we give aspirin anyway. Alternatively, we need to have a backup from the obstetrician organisation in the province or at the district level first. I think they are very open (for any suggestion) because our maternal mortality rate is high” (GP 1, Interview 1)*
	Champions involvement	*“I think later for your further study, you need to involve consultations with obstetrician consultant or organisation. This case (HDP management) in primary care is under the obstetricians organisation responsibility. Usually, the highest resistance is within the obstetrician organisation. If any women died from preeclampsia in primary care, those will also be audited by obstetricians” (Interview 5, Obstetrician)*
	More training and education tools	*“Nurse 1: I would suggest more posters and training provided to the clinic or maybe the big one so that pregnant women can easily read it* *Nurse 2: Something like dangerous signs for preeclampsia to increase awareness for the patients”. (Nurse 1 and 2, FG 10)*

#### Empowerment

This theme represents the successful impacts of the pathways, such as the providers’ positive experience and the sense of practice improvement by using the pathways in their practice. Almost all primary care provider participants in the FGs/interviews (n = 37, 92% of the interviewed primary care participants) expressed that content of the pathways was easy to follow and that it provided more comprehensive guidance on HDP management. The intervention toolkits such as posters, risk-factor checklists, and stickers were also able to remind the providers of important HDP recommendations. The providers also claimed that the current Indonesian ANC guidelines provided limited guidance on the follow-up procedures for women with preeclampsia risk factors, and that there had been no further firm guidance on HDP management in the Puskesmas or from the local health office before the study.

Many primary care provider participants (n = 37, 92% of the interviewed primary care participants) also felt that the pathways enabled them to improve their HDP management, via means such as calcium supplementation and low-dose aspirin prescriptions. The providers were now also able to request appropriate blood examinations for preeclampsia monitoring, whereas previously, they only could monitor the women’s condition mainly with blood pressure measurement and dipstick proteinuria tests. Some participants (n = 17) then went further, saying that the pathways helped them to practice optimally under the JKN insurance regulations, which required them to manage patients appropriately in primary care.

Some notes from the observations also support this theme. One woman presented to Puskesmas 3 in her 35^th^ week of pregnancy and had impending preeclampsia symptoms of edema in both legs, tremors, and ‘pins and needles’ sensations in her fingers. There was considerable confusion amongst the GPs and midwives about whether to refer the patient immediately or ask her to consult a specialist at her next hospital appointment. The GPs then decided to follow instructions in the pathway and requested kidney and liver function tests, which showed abnormal creatinine results. The patient was then immediately transferred to a hospital and safely gave birth two days after the admission.

#### Hierarchy

Hierarchical interprofessional relationships between the providers were a prominent challenge of the pathway implementation that was mentioned in almost all interviews and FGs with clinician participants. Firstly, there were implicit professional boundaries that existed in the Puskesmas. The ANC visits in Puskesmas were conducted mainly by midwives. Women were only occasionally referred to GPs, and rarely saw nurses during their routine visits. They would only see GPs at their first ANC visit, or if they had morbidities, as only GPs could prescribe medications for pregnancy or refer the women to hospitals.

Secondly, there was a sense of imbalanced hierarchical positions between GPs and patients in the Puskesmas during the pathway implementation. At the interviews, many providers mentioned that patients were very compliant with their advice, including undergoing laboratory examinations and taking medicine prescriptions. However, some patients expressed concerns to the midwives on receiving the medications but avoided discussing them with their GPs. A woman in Puskesmas 2 was anxious about her prescription, as she thought she was asymptomatic of pregnancy hypertension. Another woman, in Puskesmas 3, was initially crying after being told that she had to be referred to a hospital immediately. She thought she was still far from her delivery, and reported that the obstetricians and GPs did not explain her preeclampsia conditions. These two women were silent during the consultation; however, they expressed their concerns outside the GP room. They finally obeyed the GPs’ advice after being further approached and calmed by midwives. Later, the woman in Puskesmas 2 agreed with the medication and the woman in Puskesmas 3 agreed to have emergency delivery at the hospital.

Thirdly, a hierarchical imbalance also existed between GPs and specialists, which affected prescriptions of HDP medications. Several GPs (n = 6) argued that they were inexperienced and were afraid of encroaching upon specialists’ authority in obstetrics cases. However, they also voiced their concerns that JKN did not permit referrals of patients if they were asymptomatic or ineligible for HDP diagnosis. The GPs then further mentioned their frequent dilemma as the obstetricians’ discharge notes were unclear, but the patients had returned to primary care for further monitoring. Concerning this situation, some obstetricians (n = 3) supported the GPs by encouraging them to perform more HDP management; but still, they stressed the importance of obstetrician consultations, suggesting that GPs should always refer their HDP patients to hospitals (Table 4).

Fourthly, hierarchical barriers were observed between clinicians and the local health office. Most of the Puskesmas staff were public employees and the local health office coordinated their employability. Some GPs expressed their hesitance to manage HDP and thus contradict the local health policy, which ordered them to refer women with any pregnancy complications to hospitals.

#### Clinical resources

Clinical resources appeared to be another barrier for the pathway implementation, even though they did not impede implementation to the degree of the interprofessional hierarchy above. It was observed that Puskesmas had adequate supplies of aspirin and resources for electronic laboratory examinations for recommendations in the pathways. However, some GPs (n = 4) mentioned that the availability of testing reagents might deplete if many women had to undertake weekly preeclampsia blood tests. They also thought that the Puskesmas might have to demand more aspirin from the health office if it were to be given to eligible women up to the 37^th^ week of pregnancy or until delivery.

#### Direction

All participants expressed positive views regarding the possibility of the scale-up study of the pathways, as it would enhance the pathways’ value and practice uniformity in the region. Before the scale-up study, some obstetricians (n = 3) suggested revising terminologies used in the pathways in order that they be congruent with the existing Indonesian diagnostic standards. It was due to this disparity in terminology that some HDP diagnoses, such as eliminating transient hypertension or masked hypertension, were not found in the Indonesian e-medical records. Some GPs and midwives further suggested advocacy for supporting policies with local health officers and involving champion obstetricians to endorse the pathways’ uptake in primary care. More educational activities and toolkits, such as teaching, training, posters, and booklets, were also suggested to increase midwives and nurses’ skills and enhance patient awareness in HDP management.

## Discussion

This study has reported an implementation process to determine the acceptability and feasibility of HDP management pathways in Indonesian primary care. The pathways are acceptable, feasible, and have empowered the providers’ practice in HDP management, despite some practical challenges of clinical resources and professional hierarchies. There are also opportunities and suggestions for the scale-up study of the pathways by involving champion obstetricians, developing supporting policies, and providing more patients educational tools.

The underpinning theoretical frameworks of MRC and PRISM [15, 16] have helped the understanding of the pathways’ implementation process. As the primary intervention, the developed HDP pathways are able to complement current Indonesian ANC standards and improve the providers’ ability in HDP management. Knowledge transfer and dissemination process of the pathways have also been facilitated by the multifaceted intervention toolkits provided in the study, including training modules, posters, and the use of a preeclampsia risk factor checklist. The JKN policy has also facilitated the pathway implementation, through its requirement that the providers should manage patients appropriately in primary care practice.

Some intrinsic characteristics of the primary care provider participants have also supported the pathway implementation. The providers were open to expanding and were willing to elevate their practice by performing more HDP management, including HDP screenings and prescribing aspirin for preeclampsia prophylaxis. They felt empowered to challenge the status quo of HDP management in primary care, which is often hampered by professional hierarchies, rigid regulations, and limited clinical resources.

Nonetheless, there are also barriers of prominent professional boundaries and hierarchy that challenge the pathway implementation in primary care; and these barriers are not uncommon for any intervention implementation in health care settings [31]. However, the hierarchical barrier is very prominent in the Indonesian context, in which the study took place [32, 33], outweighing other classical barriers of implementation in LMICs, such as limited clinical resources and facilities [34, 35]. Nurses and midwives in Indonesia are usually positioned at a lower hierarchical level than GPs, and GPs often feel inferior to and less qualified than specialists [36]. The GPs’ confidence to apply recommendations in the pathways can easily crumble after receiving unsupportive policies from or having demotivating experiences with obstetricians, who are perceived as the ultimate experts in maternal health [37].

The power imbalance between clinicians described above may have resulted from the disparities in clinicians’ respective levels of training. Practicing nurses or midwives are usually diploma graduates in nursing or midwifery [38], and Indonesian GPs are medical doctor (MD) graduates without any further postgraduate training. However, to practice as specialists, the MD graduates have to undertake a further three to four years of specialty training in hospitals [38, 39]. Therefore, many GPs, nurses, and midwives often feel less capable, less respected and/or less trusted than specialists, and often receive less acknowledgement from specialists or patients [40, 41].

In addition, some of our findings also indicate a sense of resignation of primary care situations that represents a system issue in Indonesian health care. Some participants expressed their clinical inertia by referring to limited clinical resources available in primary care, even though some medicines and reagents were affordable and available in Puskesmas. Therefore, if the Indonesian government desperately wants to accelerate maternal mortality reduction, the health policies must consider the long-term benefits of HDP management in primary care by reinforcing policies to support primary care practice [42].

### Strengths and limitations

Key strengths of the study are that it has a rigorous study design, and that it applied multifaceted intervention toolkits to optimise the participants’ exposure to the pathways [43–45]. The evaluation of the pathway implementation also involved triangulation of clinical audits, observations, and FGs/interviews to allow in-depth analysis and to complement findings from each evaluation method [46]. The number of participants in the study was considerable and provided similar implementation evaluations, which optimises our confidence in the data saturation. The FGs were also conducted with participants in the same professional group to minimise biases and power imbalance of dominant participants [47]. The first author, who is an Indonesian GP researcher, conducted the dissemination seminars and all FGs/interviews. Her experience of working with and interviewing primary care providers has also enabled candid responses from the participants during the FGs/interviews.

However, due to the short implementation period, not many patient participants provided their contact for interview arrangements, which limited the opportunities to listen to their evaluation for the pathways [12]. Lastly, the pathways were only implemented in three Puskesmas in Yogyakarta province, which may not represent situations in private practices or other Indonesian provinces.

### Implications for practice

This study has shown that the developed HDP management pathways are feasible and acceptable for their implementation in Indonesian primary care. The pathways can be used to inform policy or to guide local health offices to improve HDP management in primary care. Results of pathway implementation in this study can also be used as to inform strategy to reduce maternal mortality in Indonesia or other LMICs with similar contexts [48, 49].

### Implications for future research

Our study has identified key barriers to the pathways’ implementation feasibility in primary care. According to the MRC guidelines [15], future research should examine strategies to minimise these barriers before conducting further study into pathways efficacy or more extensive implementation studies to enhance the pathways’ values in primary care practice. Professional boundaries and hierarchical barriers as the prominent challenge of pathway implementation could potentially be minimised by providing interprofessional training that enables knowledge sharing between the providers [50]; or, as mentioned by the participants in this study, by involving champion obstetricians and advocating supporting policies to endorse the pathway uptakes in primary care [15, 51].

## Conclusion

This study has determined the acceptability and feasibility of the developed HDP management pathways in Indonesian practice, and has provided foundations for further research in primary care. Notable challenges of the pathway implementation have also been identified, such as professional boundaries and hierarchy among the providers. Further investigations to minimise the implementation barriers are desired before conducting scale-up implementation of the pathway in primary care.

## Supplementary Information

**Additional file 1: Supplementary Table 1**. Guiding questions used in the evaluation interviews/focus groups.

**Additional file 2.** Reporting checklist for quality improvement study.

## Data Availability

Raw materials in this study contain confidential and private information. They are not suitable to share beyond the research team.

## Abbreviations

ANC: Antenatal care
GPs: General practitioners
HDP: Hypertensive disorders of pregnancy
LMICs: Low-and middle-income Countries
JKN: *Jaminan Kesehatan Nasional* (Indonesian national public insurance)
PLS: Plain Language Statement
Posyandu: Pos Pelayanan Terpadu (Community outreach program)
Puskesmas: *Pusat Kesehatan Masyarakat* (Indonesian public primary care clinic)
SOP: Standard Operating Procedures
