# Supplementary material for: Challenging the status quo: results of an acceptability and feasibility study of hypertensive disorders of pregnancy (HDP) management pathways in Indonesian primary care
Source: BMC Pregnancy Childbirth. 2021 Jul 14;21:507. doi: 10.1186/s12884-021-03970-8 (PMC8278644; doi:10.1186/s12884-021-03970-8)
Supplement: Supplementary file 1 — Additional file 1: Supplementary Table 1. Guiding questions used in the evaluation interviews/focus groups. [file 12884_2021_3970_MOESM1_ESM.docx]

Supplementary Table 1. Guiding questions used in the evaluation interviews/focus groups

| PRISM domains | Guiding questions used in the interviews/focus groups | | | |  |
| --- | --- | --- | --- | --- | --- |
|  | Primary care providers  (GPs, nurses and midwives) | Specialists | Local health officers | Patients | |
| Intervention | - What do you think about the pathway content? - What advantages do the pathways have compared to the existing guidelines? - What do you think about evidence or recommendations in the pathways? | - What do you think about the pathway content? - What advantages do the pathways have compared to the existing guidelines? - What do you think about evidence or recommendations in the pathways? | - What do you think about the pathway content? - What do you think of any policy needed for the pathway implementation in primary care? - What do you think about the pathways compared to your office's long-term goal? - What advantages do the pathways have compared to the existing guidelines? | - - Please tell me your experience of seeing GPs/midwives/nurse in Puskesmas over the past month?   - What are things going well during the consultation?   - What are the things that need to be improved during the consultation? | |
| Recipients and piloting process | - What do you think about the pathway implementation in your practice? - What are the things that went well during the implementation? - What are the things that need to be improved? | - What are the things in the Puskesmas that went well? - What are the things in the Puskesmas that need to be improved? | - What are supports or policies from your organisation that are beneficial for the pathway implementation in primary care settings? | - What are things going well during the consultation? - What are the things that need to be improved during the consultation? | |
| External environment | - How was your experience with patients during the implementation of the pathways? - What do your patients feel when they receive your treatment? - What are further supports needed for the pathway implementation? - In your opinion, what does the insurance/local health officer think of the pathways? | - What do you think about the referral from Puskesmas over the past month? - What are the things going well with the referral? - In your opinion, what probably do the insurance or local health officer think of the pathways? | - What are supports or policies from the provincial health office that are beneficial for the pathway implementation in primary care? | - What do you think if similar treatments are applied to other pregnant women in a wider region? | |
| Implementation and sustainability | - What do you think if the pathways are applied to wider participants? (such as for future implementation or controlled studies?) - Would you please provide more suggestions for further implementation or controlled studies (patient recruitment)? | | | |  |
