# Supplementary material for: Challenging the status quo: results of an acceptability and feasibility study of hypertensive disorders of pregnancy (HDP) management pathways in Indonesian primary care
Source: BMC Pregnancy Childbirth. 2021 Jul 14;21:507. doi: 10.1186/s12884-021-03970-8 (PMC8278644; doi:10.1186/s12884-021-03970-8)
Supplement: Supplementary file 2 — Additional file 2. Reporting checklist for quality improvement study. [file 12884_2021_3970_MOESM2_ESM.docx]

# Reporting checklist for quality improvement study.

Based on the SQUIRE guidelines.

## SQUIRE Checklist of paper, tittle: *Challenging the status quo:* results of an acceptability and feasibility study of hypertensive disorders of pregnancy (HDP) management pathways in Indonesian primary care

|  |  | Reporting Item | Page Number |
| --- | --- | --- | --- |
| **Title** |  |  |  |
|  | [#1](https://www.goodreports.org/squire/info/#1) | Indicate that the manuscript concerns an initiative to improve healthcare (broadly defined to include the quality, safety, effectiveness, patient centeredness, timeliness, cost, efficiency, and equity of healthcare) | Page 1. Title |
| **Abstract** |  |  |  |
|  | [#02a](https://www.goodreports.org/squire/info/#02a) | Provide adequate information to aid in searching and indexing | Page 2. Abstract |
|  | [#02b](https://www.goodreports.org/squire/info/#02b) | Summarize all key information from various sections of the text using the abstract format of the intended publication or a structured summary such as: background, local problem, methods, interventions, results, conclusions | Page 2. Abstract |
| **Introduction** |  |  |  |
| Problem description | [#3](https://www.goodreports.org/squire/info/#3) | Nature and significance of the local problem | Page 3 Background |
| Available knowledge | [#4](https://www.goodreports.org/squire/info/#4) | Summary of what is currently known about the problem, including relevant previous studies | Page 3 Background |
| Rationale | [#5](https://www.goodreports.org/squire/info/#5) | Informal or formal frameworks, models, concepts, and / or theories used to explain the problem, any reasons or assumptions that were used to develop the intervention(s), and reasons why the intervention(s) was expected to work | Page 3. Background Par. 3, Page 4. Methods (Theoretical Framework). |
| Specific aims | [#6](https://www.goodreports.org/squire/info/#6) | Purpose of the project and of this report | Page 3. Background Par. 3 |
| **Methods** |  |  |  |
| Context | [#7](https://www.goodreports.org/squire/info/#7) | Contextual elements considered important at the outset of introducing the intervention(s) | Page 4. Study setting |
| Intervention(s) | [#08a](https://www.goodreports.org/squire/info/#08a) | Description of the intervention(s) in sufficient detail that others could reproduce it | Page 5 Intervention |
| Intervention(s) | [#08b](https://www.goodreports.org/squire/info/#08b) | Specifics of the team involved in the work | Page 4-5. Design and intervention |
| Study of the Intervention(s) | [#09a](https://www.goodreports.org/squire/info/#09a) | Approach chosen for assessing the impact of the intervention(s) | Page 4. Design, Page 8-9 Data collection |
| Study of the Intervention(s) | [#09b](https://www.goodreports.org/squire/info/#09b) | Approach used to establish whether the observed outcomes were due to the intervention(s) | Page 5. Design, Page 6-7 Data collection |
| Measures | [#10a](https://www.goodreports.org/squire/info/#10a) | Measures chosen for studying processes and outcomes of the intervention(s), including rationale for choosing them, their operational definitions, and their validity and reliability | Page 6-7 Data collection |
| Measures | [#10b](https://www.goodreports.org/squire/info/#10b) | Description of the approach to the ongoing assessment of contextual elements that contributed to the success, failure, efficiency, and cost | Page 6-7 Data collection |
| Measures | [#10c](https://www.goodreports.org/squire/info/#10c) | Methods employed for assessing completeness and accuracy of data | Page 6-7 Data collection |
| Analysis | [#11a](https://www.goodreports.org/squire/info/#11a) | Qualitative and quantitative methods used to draw inferences from the data | Page 6-7. Data analysis |
| Analysis | [#11b](https://www.goodreports.org/squire/info/#11b) | Methods for understanding variation within the data, including the effects of time as a variable | Page 4. Design and Page 7. Data analysis |
| Ethical considerations | [#12](https://www.goodreports.org/squire/info/#12) | Ethical aspects of implementing and studying the intervention(s) and how they were addressed, including, but not limited to, formal ethics review and potential conflict(s) of interest | Page 17. Ethics approval and consent to participate. |
| **Results** |  |  |  |
|  | [#13a](https://www.goodreports.org/squire/info/#13a) | Initial steps of the intervention(s) and their evolution over time (e.g., time-line diagram, flow chart, or table), including modifications made to the intervention during the project | Page 8-13. Results  Figure 4, Table 2,3 |
| p | [#13b](https://www.goodreports.org/squire/info/#13b) | Details of the process measures and outcome | Page 8-13. Results |
|  | [#13c](https://www.goodreports.org/squire/info/#13c) | Contextual elements that interacted with the intervention(s) | Page 8-13. Results |
|  | [#13d](https://www.goodreports.org/squire/info/#13d) | Observed associations between outcomes, interventions, and relevant contextual elements | Page 8-13. Results |
|  | [#13e](https://www.goodreports.org/squire/info/#13e) | Unintended consequences such as unexpected benefits, problems, failures, or costs associated with the intervention(s). | Page 8-13. Results |
|  | [#13f](https://www.goodreports.org/squire/info/#13f) | Details about missing data | Page 15. Strength and limitation |
| **Discussion** |  |  |  |
| Summary | [#14a](https://www.goodreports.org/squire/info/#14a) | Key findings, including relevance to the rationale and specific aims | Page 13-14. Discussion Par. 1-3 |
| Summary | [#14b](https://www.goodreports.org/squire/info/#14b) | Particular strengths of the project | Page 13-14. Discussion Par. 1-3 and Page 15. Strength and limitation |
| Interpretation | [#15a](https://www.goodreports.org/squire/info/#15a) | Nature of the association between the intervention(s) and the outcomes | Page 13-14. Discussion Par 1-5 |
| Interpretation | [#15b](https://www.goodreports.org/squire/info/#15b) | Comparison of results with findings from other publications | Page 13-15. Discussion Par 4-5 |
| Interpretation | [#15c](https://www.goodreports.org/squire/info/#15c) | Impact of the project on people and systems | Page 13-15. Discussion Par 4-6 and Page 16 Implications for practice and further research |
| Interpretation | [#15d](https://www.goodreports.org/squire/info/#15d) | Reasons for any differences between observed and anticipated outcomes, including the influence of context | Page 13-15. Discussion |
| Interpretation | [#15e](https://www.goodreports.org/squire/info/#15e) | Costs and strategic trade-offs, including opportunity costs | Page 15. Discussion Par 6 |
| Limitations | [#16a](https://www.goodreports.org/squire/info/#16a) | Limits to the generalizability of the work | Page 15. Strength and limitation |
| Limitations | [#16b](https://www.goodreports.org/squire/info/#16b) | Factors that might have limited internal validity such as confounding, bias, or imprecision in the design, methods, measurement, or analysis | Page 15. Strength and limitation |
| Limitations | [#16c](https://www.goodreports.org/squire/info/#16c) | Efforts made to minimize and adjust for limitations | Page 15. Strength and limitation |
| Conclusion | [#17a](https://www.goodreports.org/squire/info/#17a) | Usefulness of the work | Page 16. Conclusion |
| Conclusion | [#17b](https://www.goodreports.org/squire/info/#17b) | Sustainability | Page 16. Implications for further research and Page 16. Conclusion. |
| Conclusion | [#17c](https://www.goodreports.org/squire/info/#17c) | Potential for spread to other contexts | Page 16. Implications for further research and Conclusion. |
| Conclusion | [#17d](https://www.goodreports.org/squire/info/#17d) | Implications for practice and for further study in the field | Page 16. Implications for practice and further research. |
| Conclusion | [#17e](https://www.goodreports.org/squire/info/#17e) | Suggested next steps | Page 16. Conclusion. |
| **Other information** |  |  |  |
| Funding | [#18](https://www.goodreports.org/squire/info/#18) | Sources of funding that supported this work. Role, if any, of the funding organization in the design, implementation, interpretation, and reporting | Page 17. Declaration (Funding). |

None The SQUIRE 2.0 checklist is distributed under the terms of the Creative Commons Attribution License CC BY-NC 4.0. This checklist can be completed online using <https://www.goodreports.org/>, a tool made by the [EQUATOR Network](https://www.equator-network.org) in collaboration with [Penelope.ai](https://www.penelope.ai)
